# Community analysis of biofilms on flame-oxidized stainless steel anodes in microbial fuel cells fed with different substrates

**DOI:** 10.1186/s12866-017-1053-z

**Published:** 2017-06-29

**Authors:** Nweze Julius Eyiuche, Shiho Asakawa, Takahiro Yamashita, Atsuo Ikeguchi, Yutaka Kitamura, Hiroshi Yokoyama

**Affiliations:** 1 Graduate School of Life and Environmental Sciences, University of Tsukuba, 1-1-1 Tennodai, Tsukuba, Ibaraki 305-8572 Japan; 2 Department of Microbiology, University of Nigeria, Nsukka, Enugu State 400241 Nigeria; 3 Faculty of Agriculture, Utsunomiya University, 350 Minemachi, Utsunomiya, 321-8505 Japan; 4 Division of Animal Environment and Waste Management Research, Institute of Livestock and Grassland Science, National Agriculture and Food Research Organization (NARO), 2 Ikenodai, Tsukuba, 305-0901 Japan

**Keywords:** Community structure, Flame oxidation, *Geobacter*, *Desulfuromonas*, Microbial fuel cell, Stainless steel anode

## Abstract

**Background:**

The flame-oxidized stainless steel anode (FO-SSA) is a newly developed electrode that enhances microbial fuel cell (MFC) power generation; however, substrate preference and community structure of the biofilm developed on FO-SSA have not been well characterized. Herein, we investigated the community on FO-SSA using high-throughput sequencing of the 16S rRNA gene fragment in acetate-, starch-, glucose-, and livestock wastewater-fed MFCs. Furthermore, to analyze the effect of the anode material, the acetate-fed community formed on a common carbon-based electrode—carbon-cloth anode (CCA)—was examined for comparison.

**Results:**

Substrate type influenced the power output of MFCs using FO-SSA; the highest electricity was generated using acetate as a substrate, followed by peptone, starch and glucose, and wastewater. Intensity of power generation using FO-SSA was related to the abundance of exoelectrogenic genera, namely *Geobacter* and *Desulfuromonas*, of the phylum Proteobacteria, which were detected at a higher frequency in acetate-fed communities than in communities fed with other substrates. Lactic acid bacteria (LAB)—*Enterococcus* and *Carnobacterium*—were predominant in starch- and glucose-fed communities, respectively. In the wastewater-fed community, members of phylum Planctomycetes were frequently detected (36.2%). Exoelectrogenic genera *Geobacter* and *Desulfuromonas* were also detected in glucose-, starch-, and wastewater-fed communities on FO-SSA, but with low frequency (0–3.2%); the lactate produced by *Carnobacterium* and *Enterococcus* in glucose- and starch-fed communities might affect exoelectrogenic bacterial growth, resulting in low power output by MFCs fed with these substrates. Furthermore, in the acetate-fed community on FO-SSA, *Desulfuromonas* was abundant (15.4%) and *Geobacter* had a minor proportion (0.7%), while in that on CCA, both *Geobacter* and *Desulfuromonas* were observed at similar frequencies (6.0–9.8%), indicating that anode material affects exoelectrogenic genus enrichment in anodic biofilm.

**Conclusions:**

Anodic community structure was dependent on both substrate and anode material. Although *Desulfuromonas* spp. are marine microorganisms, they were abundant in the acetate-fed community on FO-SSA, implying the presence of novel non-halophilic and exoelectrogenic species in this genus. Power generation using FO-SSA was positively related to the frequency of exoelectrogenic genera in the anodic community. Predominant LAB in saccharide-fed anodic biofilm caused low abundance of exoelectrogenic genera and consequent low power generation.

## Background

The microbial fuel cell (MFC) is a bioelectrochemical device that directly generates electricity by oxidation of organic matter under anaerobic conditions [1–3]. Anodes based on carbon materials such as carbon cloth, carbon fiber, carbon nanotubes, and graphene have been widely used in MFCs as a result of their specific surface area, chemical stability, and biocompatibility [4]. However, metal and metal-oxide-based anodes are not used frequently because of low electrical output. Recently, a new method that oxidizes the surface of stainless steel anodes (SSA) with flame has been reported to improve current output in bioelectrochemical systems [5] and power generation in MFCs [6]. The maximum power density using flame-oxidized (FO) SSA in MFCs was 24% higher than that of a common carbon-based electrode, carbon-cloth anode (CCA) [6]. FO-SSA can be easily prepared, and stainless steel is inexpensive, highly conductive, and chemically and physically strong. Flame oxidation leads to the formation of Fe-oxide nanoparticles on the SSA surface, and the particles formed have been suggested to gather exoelectrogenic and Fe-oxide reducing bacteria onto the surface [6].

Microorganisms adhere to the anodic surface in MFCs, and some of the bacterial species, called exoelectrogenic bacteria [7], among the adherent microorganisms can transfer electrons from organic matter to the anode, via several electron-transfer pathways, such as direct transfer through membrane-bound c-type cytochrome [8], transfer using conductive pili [9], and self-mediated transfer via endogenous redox-active metabolites [10]. *Geobacter* species are well-characterized exoelectrogenic bacteria in the phylum Proteobacteria [11], and are Fe(III)-oxide reducing bacteria, found in a variety of anoxic subsurface environments [12]. *Geobacter* has been demonstrated to generate current by pathways involving direct electron transfer and pili [8, 9]. Many exoelectrogenic bacteria including *Geobacter* can directly produce current from acetate without cooperation from other bacteria [13]. However, when complex substrates, such as glucose, starch, cellulose, proteins, and organic matter present in wastewater, are fed to MFCs, non-exoelectrogenic bacteria decompose them into simple substrates that are available to exoelectrogenic bacteria. The non-exoelectrogenic bacteria are crucial for efficient electricity generation from complex substrates.

As the performance of MFCs depends on the kind of microorganisms present in the anodic biofilm, it is important to understand the mechanism of community-structure formation. The substrate preference and community structure for the biofilm developed on carbon-based anodes have been well studied [13–15]. However, as the flame-oxidation technique was developed recently, the dependency of community structure on the type of substrate has not been examined for FO-SSA. The kinds of exoelectrogenic bacteria preferentially enriched in the FO-SSA biofilm are unknown. Next-generation sequencing technology is a powerful tool for analyzing bacterial community structure at extremely fine resolution [16]. High-throughput sequencing analyzes several million reads for the 16S rRNA gene, by which slight differences between bacterial community structures can be detected. In the present study, the communities on FO-SSA fed with defined substrates and livestock wastewater were investigated by high-throughput sequencing. To analyze the effect of the anode material, the community formed on the standard carbonaceous electrode, CCA, fed with acetate was also examined.

## Methods

### MFC operation and power density

FO-SSA was made from a 0.2-mm thick mesh (100 mesh, SUS304, 100-μm wire diameter) by flame oxidation as described previously [6]. The FO-SSA (4 cm × 80 cm) was folded and placed in a single-chambered air-cathode MFC reactor [17]. The MFC was cubic in shape with an inner volume of 125 mL (5 cm × 5 cm × 5 cm), fabricated with 0.8 cm thick polycarbonate resin. A carbon-paper cathode containing 0.5 mg/cm^2^ of Pt catalyst was placed on one side of the MFC. The reactor configuration and electrode sizes were the same as the membrane-less MFCs used in the previous study [6]. Livestock wastewater and a basal medium supplemented with 2 g/L glucose or soluble starch were fed to the MFCs. The basal medium contained per liter: 1 g meat extract, 0.3 g urea, 0.6 g NaH_2_PO_4_·2H_2_O, 2 g NaHCO_3_, 0.12 g NaCl, 0.05 g KCl, 0.03 g CaCl_2_·2H_2_O, and 0.05 g MgSO_4_·7H_2_O. Wastewater with a 1000–1500 mg/L biochemical oxygen demand was collected from the cattle and swine barns at the Institute of Livestock and Grassland Science, NARO (Tsukuba-city, Ibaraki, Japan). Activated sludge sampled at a wastewater-treatment plant in the Institute was inoculated into all the MFCs as the seed sludge. The MFCs were operated at 30 °C in a fed-batch mode. The MFCs were connected to a 4.3 kΩ external resistor, and the resistance value was decreased stepwise to 1.1 kΩ and 0.36 kΩ during operation. For comparison, a CCA with a size of 5 cm × 5 cm was placed opposite the cathode in the cubic MFC reactor, and the activated sludge was inoculated. The MFC equipped with CCA was fed with basal medium containing 2 g/L sodium acetate, and was operated in the same manner as the MFCs with FO-SSA. After accumulation culture for 6–8 weeks, the electrical power of the MFCs was determined using a potentiostat/galvanostat, as described previously [6]. The power density was normalized with respect to the projected-cathode area (m^2^).

### High-throughput sequencing and taxonomic assignment

Next-generation sequencing was performed with the MiSeq Illumina sequencing platform (Illumina Inc., CA, USA) targeting the V3–V4 region of the 16S rRNA gene [16]. The anodes were thoroughly washed with distilled water and then cut into small pieces. Genomic DNA of the biofilms was extracted from the anode pieces using an UltraClean™ Soil DNA Isolation kit (Mo Bio Laboratories, Carlsbad, CA, USA). Libraries were constructed through PCR using the primers with the Illumina overhang adapter sequences, 357F (5′-TCG TCG GCA GCG TCA GAT GTG TAT AAG AGA CAG CCT ACG GGN GGC WGC AG-3′) and 802R (5′-GTC TCG TGG GCT CGG AGA TGT GTA TAA GAG ACA GTA CNV GGG TAT CTA ATC C-3′), as specified by the manufacturer’s instructions. PCR products were purified using an AMPure XP kit (Beckman Coulter, Miami, FL, USA) and were quantified for equimolar pooling by using a NanoDrop™ spectrophotometer (Thermo Fisher Scientific, Waltham, MA, USA). The libraries were sequenced on a 300PE MiSeq run, and image analysis, base calling, and data quality assessment were performed with the MiSeq Reporter software (Illumina). Paired-end read data exported in FASTQ format were joined and quality-checked with the Quantitative Insights Into Microbial Ecology (QIIME) software ver. 1.8 [18]. The joined read sequences were clustered into operational taxonomic units (OTUs) at a similarity threshold of 97% by using the Uclust method [19] with the Greengenes reference database [20] and the QIIME script “pick_open_reference_otus.py”. Singletons were removed with the script. Representative sequences were aligned using PyNAST [21], and a phylogenetic tree was constructed. The taxonomic classification and alpha and beta diversities were computed using QIIME. The taxonomic assignment of the major OTUs was checked using BLAST and Classifier [22]. The beta diversity was calculated using a weighted UniFrac distance matrix [23], and the result was visualized using a principal coordinate (PCo) plot. The phylogenetic tree, combined with the heat map, was calculated by the unweighted pair-group method using arithmetic averages (UPGMA) using MEGA4 [24]. The sequencing data of representative OTUs and all reads were deposited in DDBJ under accession numbers LC209094–LC209102 and DRR090365-DRR090369 (Sequence Read Archive), respectively.

## Results and discussion

### Electricity generation from different substrates

**Fig. 1:**
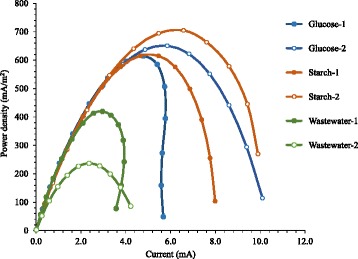
Power density of the MFCs equipped with FO-SSA using different substrates as fuel. Two reactors were operated for each substrate

To examine the effect of substrates on production of current by FO-SSA, two kinds of defined substrate, glucose and starch, and livestock wastewater were fed to the MFCs. Two MFC reactors were operated for each substrate, and power density was measured once per reactor. The MFCs fed with starch and glucose generated similar levels of maximum power density, 615–704 mW/m^2^ (average 660 mW/m^2^) and 614–650 mW/m^2^ (average 632 mW/m^2^), respectively (Fig. 1 and Table 1). Livestock wastewater consists of complex substrates and large amounts of non-electrogenic microorganisms that compete with exoelectrogenic bacteria for substrate utilization. Thus, compared with the values for MFCs with starch and glucose, the maximum power density of the MFCs with wastewater was approximately half, 236–419 mW/m^2^ (average 328 mW/m^2^). Furthermore, cathode performance considerably affects the power generation of MFCs; consequently, heterogeneity in power generation was observed between the two MFCs fed with the same substrate owing to the slightly non-uniform coating of the Pt-catalyst on the cathode surface.

**Table 1:** Experimental conditions for different anodic biofilms in the microbial fuel cells and their power output

	GM-FO-SSA	SM-FO-SSA	WW-FO-SSA	AM-FO-SSA	AM-CCA
Substrate	Glucose	Soluble starch	Livestock wastewater	Acetate	Acetate
Anode material	Flame-oxidized stainless steel mesh (SUS304)	Flame-oxidized stainless steel mesh (SUS304)	Flame-oxidized stainless steel mesh (SUS304)	Flame-oxidized stainless steel mesh (SUS304)	Carbon cloth
Inoculum	Activated sludge	Activated sludge	Activated sludge	Activated sludge	Activated sludge
Maximum power density (mW/m^2^)	632	660	328	1,063^a^	310

*Abbreviations*: *FO-SSA* flame-oxidized stainless steel anode, *CCA* carbon-cloth anode, *AM* acetate medium, *SM* starch medium, *GM* glucose medium, *WW* wastewater

^a^Reported previously [6]

We previously reported that the maximum power density using FO-SSA was 1063 mW/m^2^ for acetate and 798 mW/m^2^ for peptone [6]. These values are higher than those observed for starch, glucose, and wastewater. Therefore, substrate type influenced the power output using FO-SSA, and the preferred substrate for the biofilm on FO-SSA was acetate, followed by peptone, then starch and glucose (equal), and finally wastewater. This substrate preference is similar to that of biofilm formed on carbon-based anodes [13]. Furthermore, these results demonstrated that generation of electricity using FO-SSA was possible from livestock wastewater, although the intensity was lower than that from defined substrates.

### Community structure of biofilm developed on FO-SSA

We previously reported the community structure on FO-SSA fed with peptone [6], but we not did examine the same for FO-SSA fed with acetate. Thus, in this study, the biofilm on FO-SSA fed with acetate, glucose, starch, and livestock wastewater was analyzed by high throughput sequencing targeting an amplified 16S rRNA gene fragment. Operational conditions for the acetate-fed MFCs with FO-SSA were the same as those for MFCs fed with other substrates. In addition, to investigate the influence of the anode material, the biofilm formed on the common carbon-based anode (CCA) fed with acetate was also analyzed; the MFC with CCA produced the maximum power density of 310 mW/m^2^. Animal manure is known to contain various kinds of useful bacteria including exoelectrogens, hydrogen-producing bacteria, and methanogens for anaerobic digestion [25, 26], and the activated sludge from animal manure treatment that was inoculated as the seed sludge into all MFCs was identical.

**Fig. 2:**
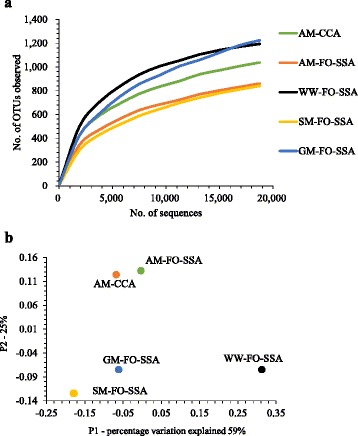
Rarefaction curves (**a**) and PCo plot (**b**) showing the relationship among the biofilm communities on FO-SSA or CCA fed with acetate medium (AM), starch medium (SM), glucose medium (GM), or livestock wastewater (WW)

In total, 239,571 sequencing reads were generated, and the reads were grouped into 3043 OTUs. The OTU distribution and alpha diversity indexes of the communities are shown in Table 2. The range of alpha diversity was 1200–2300 for Chao1 richness and 5.6–7.9 for Shannon’s diversity index. While Good’s coverage was more than 0.98 in all the communities, none of the rarefaction curves reached a plateau (Fig. 2a). In the beta diversity analysis, the community structure on FO-SSA with acetate was the closest to that of CCA with acetate in the principal coordinate (PCo) plot (Fig. 2b), and was distinct from the other communities, suggesting that the overall structures of the acetate-fed communities on FO-SSA and CCA were similar. The community on FO-SSA with starch was close to that of glucose in the plot. The similarity between the community structures for starch and glucose seems to be reasonable, since glucose is a product of starch hydrolysis.

**Table 2:** Number of reads and alpha diversity index for the anodic-biofilm communities in microbial fuel cells fed with different substrates

Sample	No. of reads	No. of OTUs	Chao1 richness	Shannon’s diversity index	Abundance-based coverage estimator	Good’s coverage
AM-CCA	86,298	1645	1951	7.475	1956	0.996
AM-FO-SSA	58,305	1249	1486	6.818	1504	0.995
SM-FO-SSA	26,190	1002	1294	5.646	1333	0.988
GM-FO-SSA	48,481	1838	2282	6.963	2323	0.989
WW-FO-SSA	18,819	1303	1513	7.84	1546	0.983

*Abbreviations*: *OUT* operational taxonomic units, *FO-SSA* flame-oxidized stainless steel anode, *CCA* carbon-cloth anode, *AM* acetate medium, *SM* starch medium, *GM* glucose medium, *WW* wastewater

**Fig. 3:**
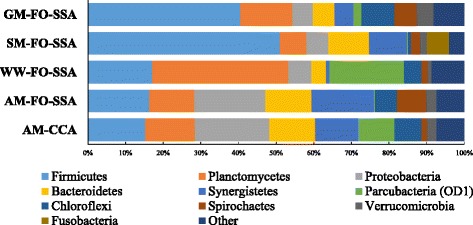
Phylum distribution of the biofilm communities on FO-SSA or CCA fed with acetate medium (AM), starch medium (SM), glucose medium (GM), or livestock wastewater (WW)

All communities were dominated by representatives of six phyla: Firmicutes, Planctomycetes, Proteobacteria, Bacteroidetes, Synergistetes, and Parcubacteria (Fig. 3). The three phyla Proteobacteria, Firmicutes, and Bacteroidetes are frequently observed in MFCs [27]. Proteobacteria including electrogenic and Fe(III)-oxide reducing bacteria such as *Geobacter* were detected at a high frequency (18.9–19.8%) in the acetate-fed communities, while the abundance of the phylum was low (5.4–6.2%) in the other communities. Firmicutes including saccharolytic anaerobes such as *Clostridium* were abundant (40.6–51.0%) in the glucose- and starch-fed communities, as compared to the other communities (15.3–17.1%). In the wastewater-fed community, the most predominant phylum was Planctomycetes (36.2%). A Planctomycetes-dominant biofilm was reported in an anodic biofilm of a bioelectrochemical system that was fed with livestock wastewater [28]. Although there are no known exoelectrogenic members in Planctomycetes, the Planctomycetes-dominant structure might be a feature of the exoelectrogenic community resulting from feeding with livestock wastewater.

### Effect of substrate and anodic material on exoelectrogenic bacteria

**Fig. 4:**
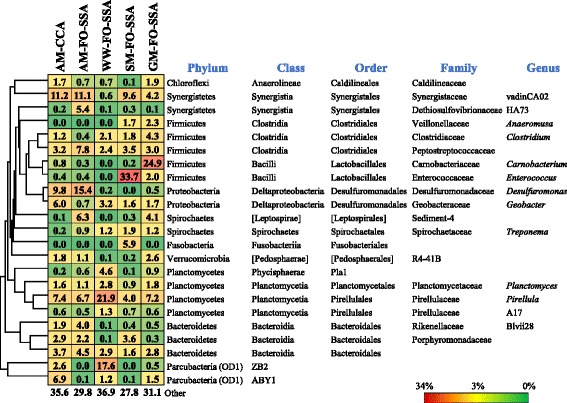
Phylogenetically clustered heat map of representative genera of biofilm communities on FO-SSA or CCA fed with acetate medium (AM), starch medium (SM), glucose medium (GM), or livestock wastewater (WW)

**Fig. 5:**
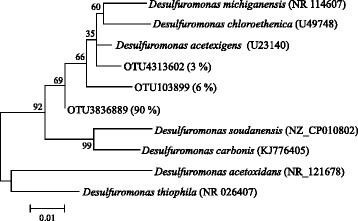
Neighbor-joining phylogenetic tree based on the 16S rRNA gene depicting the relationship between OTUs and *Desulfuromonas* species. The percentages represent the number of reads assigned to the OTUs per number of reads assigned to the genus in the anodic communities. Numbers on major branch points indicate the percentage of 500 bootstrap replicates. The *scale bars* represent a 1% difference in the DNA sequences

Two exoelectrogenic genera, *Geobacter* and *Desulfuromonas*, were detected by the genus-level analysis (Fig. 4). Both genera belong to the same order Desulfuromonadales within the phylum Proteobacteria. *Desulfuromonas* was abundant (15.4%) in the community on FO-SSA fed with acetate, while *Geobacter* was minor (only 0.7%). In the community on CCA with acetate, both *Geobacter* and *Desulfuromonas* were observed at similar abundant frequencies (6.0–9.8%). This result indicates that exoelectrogenic genus enrichment in anodic biofilm is dependent on the anode material used. *Desulfuromonas* species are marine anaerobes that reduce sulfur [29] and Fe(III) oxide [30] coupled with acetate oxidation. *Desulfuromonas acetoxidans* and ‘*Desulfuromonas soudanensis’* produce electric current using acetate [31, 32]. *Desulfuromonas* is frequently enriched in anodic biofilms using saline medium due to its halophilic characteristics [33], but is infrequently enriched with a conventional low-salt medium. *Geobacter* is a common exoelectrogenic genus that is enriched with low-salt medium. Although the low-salt medium was used in this study, it is interesting to note that *Desulfuromonas* was more predominant than *Geobacter* in the community on FO-SSA fed with acetate. OTUs affiliated with *Desulfuromonas* showed 91–96% identity in DNA sequence to *D. acetoxidans* and *D. soudanensis* (Fig. 5). The bacteria corresponding to the OTUs are inferred to be novel exoelectrogenic species in the genus *Desulfuromonas*, since the bacteria are not halophilic, which is an important feature of the genus. The major difference between FO-SSA and CCA is the presence or absence of Fe oxide on the surface. The *Desulfuromonas* bacteria could apparently take advantage of Fe oxide more proficiently, resulting in greater abundance in comparison with *Geobacter* in the acetate-fed community on FO-SSA.

The electrogenic genera *Geobacter* and *Desulfuromonas* were also detected in the communities on FO-SSA fed with glucose, starch, and wastewater, but the frequency was low (0–3.2%). This result is consistent with the data of low power output by the MFCs fed with these substrates. The predominant genera in the communities from feeding with starch and glucose were *Enterococcus* (33.7%) and *Carnobacterium* (24.9%), respectively. They are lactic acid bacteria (LAB) that produce lactic acid from saccharides. OTUs affiliated with *Enterococcus* showed 99% identity to the amylolytic bacterium *Enterococcus faecium* that produces lactic acid directly from starch [34]. As LAB are fermentative bacteria, current production is not needed for their growth in MFCs. *Geobacter sulfurreducens* was reported to produce current from lactate, but the intensity was lower than that from acetate [35]. Current production from lactate is not reported for the other *Geobacter* and *Desulfuromonas* species, while several *Geobacter* species oxidize lactate coupled with Fe(III)-oxide reduction [36]. Generally, acetate is thought to be a more preferred substrate than lactate for current production in MFCs [13, 15]. These observations suggest that the exoelectrogenic bacteria cannot grow fast in the communities fed with glucose and starch, since the lactate produced by *Carnobacterium* and *Enterococcus* is not the best substrate for them. In the community on FO-SSA fed with peptone [6], *Geobacter* was detected at a high frequency (8.8–9.2%), while the frequency of *Desulfuromonas* was low. A putative non-saccharolytic genus that produced acetate from amino acids was most predominant (>30%) in the peptone-fed community. The acetate produced by the genus is thought to serve as the fuel for current production by *Geobacter*, and thus the power output of the peptone-fed MFCs could be higher than that of MFCs fed with glucose and starch. The results of the present study showed that the community structure on FO-SSA depended on the substrate used, and that the intensity of power generation using FO-SSA was positively correlated with the abundance of exoelectrogenic bacteria in the anodic community.

## Conclusions

The community structure of the anodic biofilm in MFCs was dependent on both the substrate and anode material used. Based on combining the results of this study with previous work [6], acetate was the most preferred substrate for the biofilm on FO-SSA. The order of substrate preference was: acetate > peptone > starch = glucose > wastewater. The intensity of power generation using FO-SSA was positively related to the abundance of exoelectrogenic genera in the phylum Proteobacteria. Novel non-halophilic and exoelectrogenic species in the genus *Desulfuromonas* were apparently present in the biofilm on FO-SSA fed with acetate. LAB became predominant in the community fed with the saccharides (glucose and starch), and the lactate produced seemed to be of low availability to the exoelectrogenic genera, resulting in low power output with these saccharide substrates.

## Abbreviations

CCA: Carbon-cloth anode
FO-SSA: Flame-oxidized SSA
LAB: Lactic acid bacteria
MFC: Microbial fuel cell
PCo: Principal coordinate
SSA: Stainless steel anode
